# Prevalence of human filovirus infections in sub-Saharan Africa: A systematic review and meta-analysis protocol

**DOI:** 10.1186/s13643-024-02626-w

**Published:** 2024-08-15

**Authors:** Christopher S. Semancik, Christopher L. Cooper, Thomas S. Postler, Matt Price, Heejin Yun, Marija Zaric, Monica Kuteesa, Nina Malkevich, Andrew Kilianski, Swati B. Gupta, Suzanna C. Francis

**Affiliations:** 1 International AIDS Vaccine Initiative, New York, NY USA; 2 Tufts University School of Medicine, 145 Harrison Avenue, Boston, MA 02111 USA; 3 International AIDS Vaccine Initiative, Vaccine Design and Development Laboratory, Brooklyn, NY USA; 4 Department of Epidemiology and Biostatistics, University of California at San Francisco, San Francisco, CA USA; 5 IAVI Human Immunology Laboratory, Imperial College London, London, UK; 6 London School of Hygiene and Tropical Medicine, London, UK

**Keywords:** Filoviruses, Viral hemorrhagic fever, Ebolavirus, Marburgvirus, Prevalence, Diagnosis, Sub-Saharan Africa

## Abstract

**Background:**

Recent outbreaks of Ebola virus disease (EVD) and Marburg virus disease (MVD) in sub-Saharan Africa illustrate the need to better understand animal reservoirs, burden of disease, and human transmission of filoviruses. This protocol outlines a systematic literature review to assess the prevalence of filoviruses that infect humans in sub-Saharan Africa. A secondary aim is to qualitatively describe and evaluate the assays used to assess prevalence.

**Methods:**

The data sources for this systematic review include PubMed, Embase, and Web of Science. Titles, abstracts, and full texts will be reviewed for inclusion by a primary reviewer and then by a team of secondary reviewers, and data will be extracted using a pre-specified and piloted data extraction form. The review will include human cross-sectional studies, cohort studies, and randomized controlled trials conducted in sub-Saharan Africa up until March 13, 2024 that have been published in peer-reviewed scientific journals, with no language restrictions. Prevalence will be stratified by pathogen, population, assay, and sampling methodology and presented in forest plots with estimated prevalence and 95% confidence intervals. If there are enough studies within a stratum, I^2^ statistics will be calculated (using R statistical software), and data will be pooled if heterogeneity is low. In addition, assays used to detect infection will be evaluated. All studies included in the review will be assessed for quality and risk of bias using the JBI Prevalence Critical Appraisal Tool and for certainty using the GRADE certainty ratings.

**Discussion:**

Accurately measuring the rate of exposure to filoviruses infecting humans in sub-Saharan Africa using prevalence provides an essential understanding of natural history, transmission, and the role of subclinical infection. This systematic review will identify research gaps and provide directions for future research seeking to improve our understanding of filovirus infections. Understanding the natural history, transmission, and the role of subclinical infection is critical for predicting the impact of an intervention on disease burden.

**Systematic Review Registration:**

In accordance with the guidelines outlined in the PRISMA-P methodology, this protocol was registered with PROSPERO on April 7, 2023 (ID: CRD42023415358).

**Supplementary Information:**

The online version contains supplementary material available at 10.1186/s13643-024-02626-w.

## Background

Filoviruses are a family of single-stranded, negative-sense RNA viruses that are a cause of viral hemorrhagic fever (VHF), and are among the most virulent pathogens in humans and nonhuman primates [1]. Filovirus outbreaks are the result of zoonotic spillover events from animal reservoirs and subsequent spread from person to person through bodily fluids [1]. The family *Filoviridae* includes eight genera: *Orthoebolavirus*, *Orthomarburgvirus*, *Cuevavirus*, *Striavirus*, *Thamnovirus*, *Dianlovirus*, *Oblavirus*, and *Tapjovirus* [2]. Of these genera, *Orthoebolavirus* and *Thamnovirus* have multiple associated species [3]. There are six *Orthoebolavirus* species, to each of which a single virus has been assigned to date: Bombali virus (BOMV, species *Orthoebolavirus bombaliense*), Bundibugyo virus (BDBV, species *Orthoebolavirus bundibugyoense*), Reston virus (RESTV, species *Orthoebolavirus restonense*), Sudan virus (SUDV, species *Orthoebolavirus sudanense*), Taï Forest virus (TAFV, species *Orthoebolavirus taiense*), and Ebola virus (EBOV, species *Orthoebolavirus zairense*) [2]. The genus *Orthomarburgvirus* includes a single species, *Orthomarburgvirus marburgense*, to which two viruses have been assigned: Marburg virus (MARV) and Ravn virus (RAVV) [2]. Diseases caused by Ebolavirus and Marburgvirus have high associated mortality rates, with case fatality rates (CFRs) ranging from 25%-90% and 24%-88%, respectively [4–6]. The recent outbreaks in 2023 in East and West Africa illustrate the urgent need to better understand the natural history of filovirus infection and the role of subclinical infection in disease transmission, as human cases of orthomarburgvirus infection had not been observed in western Africa prior to 2022 [7].

Since the first outbreak of MARV in 1967, there have been numerous reported human outbreaks of filovirus disease globally, of which most have taken place in sub-Saharan Africa [2, 8, 9]. The 2014–2016 EBOV outbreak was the largest of these, killing 11,310 of 28,616 cases in Guinea, Liberia, and Sierra Leone, followed by a 2018–2019 EBOV outbreak in the Democratic Republic of the Congo, which killed 1,074 of the 1,604 cases [8]. Some outbreaks of orthoebolaviruses and orthomarburgviruses have impacted countries outside of sub-Saharan Africa (including in Europe, Asia, and North America). All of these outbreaks originated in sub-Saharan Africa [2]. Members of the filovirus genera *Cuevavirus*, *Striavirus*, *Thamnovirus*, and *Dianlovirus* have not been shown to infect humans to date [2].

The size, duration, and case fatality rates (CFRs) of outbreaks have varied substantially depending on the success of the response and the virus [10]. Thus, control of filovirus outbreaks requires a concert of activities, including infection prevention and control, evidence-based clinical care, disease surveillance and contact tracing, good laboratory services, safe burials, and social mobilization. Therefore, outbreak control requires tremendous organization, community engagement, and resources. Vaccines offer a means of prevention and control of outbreaks. There are currently two licensed vaccines against EBOV: ERVEBO® (Merck) and Zabdeno and Mvabea (Johnson & Johnson). ERVEBO® affords protection after single-dose administration with approximately 97.5% efficacy, whereas Zabdeno and Mvabea are administered consecutively in a prime-boost vaccination regimen with approximately 53% efficacy [11]. There are no licensed vaccines for other orthoebolaviruses or for any orthomarburgviruses; however, as of 2023, there are multiple vaccine candidates in development against MARV and SUDV, including candidates built on the same platform as the licensed ERVEBO® vaccine [12–15]. A successful strategy for deploying an efficacious vaccine to prevent or control outbreaks must be informed by an understanding of the natural history of the infection and transmission of the virus.

While orthoebolaviruses and orthomarburgviruses are not considered to be endemic, and infections can result in severe disease, there is evidence of asymptomatic or subclinical infection. Although relatively little is known about filovirus reservoirs, among suspected filovirus reservoirs are chimpanzees, fruit bats, forest antelope, and guinea pigs, which makes eradication very difficult [16]. A cross-sectional study by Glynn and colleagues reported that 2.6% of asymptomatic contacts of Ebola virus-positive individuals tested positive for antibodies to Ebola virus by a newly validated anti-glycoprotein IgG capture assay, as compared to 12.0% of symptomatic contacts of Ebola virus-positive individuals in Sierra Leone (with symptoms possibly caused by something besides a filovirus) [17]. Another cross-sectional study by Leroy and colleagues reported that 46% of asymptomatic close contacts of EBOV cases were seropositive in Gabon [18]. Subclinical infections, or even infections causing mild or moderate disease that are not recognized as Ebola or Marburg, could revise our understanding of natural history and transmission, and this understanding may contribute to the design of interventions to prevent or control these infections.

Importantly, studies of human filovirus prevalence do not typically provide precise estimates. Two recent filovirus antibody seroprevalence reviews reported wide ranges of estimates for filovirus seroprevalence. A systematic review by Bower and Glynn reported estimates for orthoebolavirus seroprevalence ranging from 0.4% to 45.8% [19]. Another systematic review by Nyakarahuka and colleagues reported estimates for orthoebolavirus seroprevalence ranging from 1.0% to 22.0% and for orthomarburgvirus seroprevalence ranging from 0.0% to 3.2% [20]. The systematic review by Bower and Glynn stratified seroprevalence results by population and exposure status, but not by species of orthoebolavirus or by assay type used for detection [19]. The systematic review by Nyakarahuka and colleagues did not stratify by population, exposure status, species, or assay used for detection [20]. Prevalence studies use a variety of different populations, sampling methods, and assays, which may contribute to the variability seen in these prior systematic reviews [17].

In this study, we propose to review published, peer-reviewed papers reporting prevalence of orthoebolavirus and orthomarburgvirus infection (through viral detection), or seroprevalence of antibodies to these viruses, in humans in sub-Saharan Africa. This review will update and expand the review of orthoebolavirus seroprevalence by Bower and Glynn in 2017 and the review of filovirus seroprevalence by Nyakarahuka and colleagues in 2016 [19, 20]. The primary question for this review is: What is the reported prevalence of orthoebolaviruses and orthomarburgviruses in sub-Saharan Africa? The secondary research question for this review is: What are the characteristics of the assays for detection of filovirus infection?

## Methods

This systematic review protocol describes the approach to reviewing and synthesizing the relevant literature to answer the research questions. The search has been designed using a Population, Intervention, Comparison, Outcome, Study Design (PICOS) framework based on the primary research question (Table 1) [21]. The data for the secondary research question will be extracted from eligible publications identified for the primary research question. This protocol adheres to the Preferred Reporting Items for Systematic Reviews and Meta-Analyses Protocol (PRISMA-P) checklist (Additional File 1) [22]. At the time of submission of this protocol for publication in December 2023, the search strategy had been finalized and completed, search result titles and abstracts had been screened, and final data extraction was under way.

**Table 1:** PICOS framework for identifying studies relevant to primary research question

**P**opulation	People living in sub-Saharan Africa
**I**ntervention	No intervention
**C**omparison	No comparison; prevalence stratified by population characteristics will be extracted
**O**utcome	Confirmed presence of virus or antibodies for EBOV, SUDV, BDBV, TAFV, RESTV, MARV, or RAVV, as measured by either viral testing (e.g., RT-PCR) or antibody testing (e.g., ELISA)
**S**tudy Design	Cross-sectional studies, cohort studies, and randomized controlled trials; no case studies or case–control studies

### Search strategy

The following databases were searched: PubMed (PubMed interface), Embase (Elsevier interface), and Web of Science (Clarivate interface). The search was performed on March 1, 2023 with no restriction on dates or language, and a second follow-up search was performed on March 13, 2024. Search terms included various combinations of the terms “Ebola,” “Marburg,” “hemorrhagic fever,” “seroprevalence,” and “epidemiology.” Specific search terms were applied in accordance with the syntax of each included database and are shown in Table 2. Furthermore, references cited by existing reviews and included papers were added individually. This search strategy was developed in collaboration with a certified librarian from the Tufts University School of Medicine Hirsh Library using the Peer Review of Electronic Search Strategies (PRESS) guidelines and Peer Assessment Form [23].

**Table 2:** Search strategies for each database

Database	Search Strategy
PubMed (PubMed interface)	“Ebolavirus” [MeSH] “Hemorrhagic Fever, Ebola” [MeSH] “Marburgvirus” [MeSH] Hemorrhagic fever*[tiab] Ebola[tiab] Ebolavirus*[tiab] Marburg virus*[tiab] Frankfurt Marburg syndrome virus[tiab] (1 OR 2 OR 3 OR 4 OR 5 OR 6 OR 7 OR 8)
	“Seroepidemiologic Studies” [MeSH:NoExp] “Epidemiologic Methods” [MeSH:NoExp] Seroprevalence*[tiab] Serology[tiab] Seroepidemiolog*[tiab] (10 OR 11 OR 12 OR 13 OR 14)
	((9) AND (15))
Embase (Elsevier interface)	‘ebola hemorrhagic fever’/exp ‘marburgvirus’/exp ‘ebolavirus’/exp ‘marburg hemorrhagic fever’/exp ebola* marburg* (1 OR 2 OR 3 OR 4 OR 5 OR 6)
	‘seroepidemiology’/exp ‘seroprevalence’/exp ‘epidemiological surveillance’/exp ‘prevalence’/exp seroepidemiol* seroprevalence* serveil* (8 OR 9 OR 10 OR 11 OR 12 OR 13 OR 14)
	[humans]/ilm
	((7) AND (15) AND (16))
Web of Science (Clarivate interface)	Ebolavirus Marburgviruses Ebola* Ebolavirus* Marburg* Marburgviruses* (1 OR 2 OR 3 OR 4 OR 5 OR 6)
	Seroepidemiol* *Prevalence* Seroprevalence* Serology Seroepidemiolog* (8 OR 9 OR 10 OR 11 OR 12)
	(TS = (7)) AND TS = (13))

### Eligibility criteria and study selection

Detailed eligibility criteria are outlined in Table 3. Eligible studies must be original primary research from peer-reviewed papers reporting the prevalence of an orthoebolavirus (SUDV, EBOV, RESTV, TAFV, or BDBV) or orthomarburgvirus (MARV or RAVV) in humans. These studies must be conducted in sub-Saharan Africa. Conference abstracts will not be eligible. Eligible study designs include cross-sectional studies, cohort studies, and randomized controlled trials. Case studies, case–control studies, and reviews will not be included, but may be reviewed to contextualize results of this systematic review. Studies using both probability and non-probability sampling will be included. Prevalence will be extracted from the non-interventional arm of randomized controlled trials. Studies will only be eligible if infection is detected with laboratory assays, but there will be no limit on the date of clinical presentation.

**Table 3:** Inclusion criteria and exclusion criteria

Inclusion Criteria	Exclusion Criteria
Human research studies	Studies with non-human animals
Cohort studies, cross-sectional studies, randomized controlled trials	Case–control studies
Studies conducted in sub-Saharan Africa	Studies conducted outside of sub-Saharan Africa
Studies on filoviruses that have previously been shown to infect humans (including orthoebolaviruses [EBOV, SUDV, TAFV, BDBV, RESTV] and orthomarburgviruses [MARV, RAVV]	Studies on filoviruses that have not caused human infection (i.e., Bombali ebolavirus and filoviruses other than orthoebolaviruses and orthomarburgviruses)
Studies from all dates until the end of the search (March 13, 2024)	Studies published after March 13, 2024
All assays for diagnosis, including RT-PCR, ELISA, or any other laboratory assay measuring antibody or filovirus antigen	
Individuals of all ages	
Individuals of all occupations and exposure statuses	

Study selection based on eligibility criteria will be carried out by one primary screener (CSS) and three secondary screeners (CC, MP, and TSP) after duplicates have been removed. All screeners will provide reasons for excluding studies. In the case of disagreement over the eligibility of a publication, a third screener will act as the tiebreaker (SCF). The Rayyan software will be used to support screening [24].

### Data extraction and quality assessment

A predefined data extraction form has been developed in Google Forms and piloted using five included papers (Additional File 2). This form includes the following variables: American Psychological Association (APA) citation; first author’s name; institutional/geographic affiliations of authors; publication year; country of study; regional description of study; study design; virus subtypes/species detected; number of participants; age range of participants; occupation of participants; other descriptions of study participants; prevalence of relevant filovirus(es) (percentage with confidence intervals); sampling method; assay type and name; and assay characteristics. Additional data will be extracted to assess the risk of bias at the study level [22]. The risk of bias will be assessed for all studies included in the review using the JBI Prevalence Critical Appraisal Tool [25]. This tool assesses the methodological quality of prevalence studies, including potential for information bias, sampling bias, coverage error, selection bias, measurement error, misclassification bias, and appropriateness of sample size. Based on assessments from the JBI Prevalence Critical Appraisal Tool, studies will be rated as ‘low risk of bias,’ ‘some concerns,’ or ‘high risk of bias.’ The data extraction will be performed by two reviewers (CSS and HY).

### Data synthesis and analysis

A PRISMA flowchart will outline the results of the search and selection process [23]. Characteristics of the included studies will be tabulated, including country of study; study design; genus and species of virus; number of participants; study population description; prevalence of relevant filovirus(es); sampling method; assay type and name; and assay characteristics. Forest plots will be constructed by extracting or calculating 95% confidence intervals around a reported point estimate, using a formula for standard error of a binomial outcome around corresponding point estimates ($\surd \frac{\left(seroprevalence\right)*(1-seroprevalence)}{population size}$) and the CONF function in Microsoft Excel (Excel 2019). Forest plots and meta-analyses (if applicable) will be constructed and performed using the functions *metaprop* and *forest* within the *meta* package in R statistical software (v4.3.0; R Core Team 2020). Prevalence estimates will be stratified by genus and species of virus, study population (e.g., healthy individuals in the general population; healthcare workers; miners), sampling methodology, and assay type. If there are at least five studies within a particular stratum, the I^2^ statistic will be calculated using the Cochran’s homogeneity test statistic and degrees of freedom for each stratum, to quantify the degree of heterogeneity among studies. Prevalence estimates will be pooled using the fixed effects model if the I^2^ statistic is less than 75% (high heterogeneity) [26]. A fixed effects model will be used in this context because stratification will occur by population, which is an indicator of mode of filovirus contact or exposure. Since the fixed effects model pools by calculating a weighted average of study-specific effect sizes, this method is most appropriate in strata where study populations have similar modes of exposure [27]. Furthermore, since random effects models give more weight to smaller and less precise studies (for which prevalence results may be less useful), the fixed effects model is a superior choice to the random effects model in this context where many small and imprecise prevalence surveys exist [27]. If a particular stratum does not have at least five component studies or has high heterogeneity, then a pooled analysis will not be calculated for that stratum and prevalence will be presented in a forest plot or described in the text. A summary table will describe the frequency, types, and quality of assays utilized for detection. Study quality will be evaluated in a table by addressing the questions included in the JBI Prevalence Critical Appraisal Tool. Within forest plots, studies will be ranked by quality, with higher quality studies (with lower risks of bias) being listed first in forest plots. A narrative synthesis will introduce and describe each of the tables.

Strength of evidence will be addressed using the Grading of Recommendations, Assessment, Development, and Evaluations (GRADE) certainty ratings [28]. Specifically, each article will be rated as one of the following certainty levels: very low, low, moderate, or high. These certainty levels will be assigned based on the following domains: potential for risk of bias, imprecision, consistency or inconsistency with other similar studies, indirectness, and publication bias. The means of assessing each domain of the GRADE framework (risk of bias, imprecision, inconsistency, indirectness, and publication bias) are outlined and examples are given for each domain in Table 4. After assigning a point value system for each domain, with very low certainty getting one point, low certainty getting two points, moderate certainty getting three points, and high certainty getting four points, an average composite score will be calculated by taking the average across all five domains of the GRADE certainty ratings, and a final GRADE score will be given after rounding the average composite score either up or down.

**Table 4:** Approach for assessing GRADE domains

GRADE Domain	Means of Assessing	Example
Risk of bias	Done in accordance with assessments from JBI Prevalence Critical Appraisal Tool	A study rated ‘high risk of bias’ will be rated as either low certainty or very low certainty in this domain, depending on extent of additional comments from reviewers
Imprecision	Considering both the sample size and by considering the validity and rigor of the methods in the study (a question from the JBI Prevalence Critical Appraisal Tool)	A study with a sample size of less than 50 individuals that also uses an outdated assay (such as immunofluorescence assay) will be rated as very low certainty in this domain
Inconsistency	Comparing the prevalence result of a given study with how closely it aligns with other prevalence results from the same stratum	If a study’s prevalence result diverges greatly from many others in the same stratum, that study will be rated as low certainty in this domain
Indirectness	Based on how well the study population is described (a question from the JBI Prevalence Critical Appraisal Tool) and whether the study’s main objective was to measure prevalence of a filovirus in humans	If a study provides detailed descriptions of the study population and a prevalence survey was the main intent of the study, that study will be rated as high certainty in this domain
Publication bias	By looking at JBI Prevalence Critical Appraisal Tool overall rating for a given study and other studies in the same stratum	If a study has itself been rated ‘high risk of bias’ and many other studies in its stratum were also rated as having concerns about bias, then the study would be rated very low certainty in this domain

## Discussion

This systematic review will provide an updated review of the prevalence of filoviruses that have caused human infection in sub-Saharan Africa, namely the orthoebolaviruses and orthomarburgviruses. This review will build on prior systematic reviews, including a 2016 review by Nyakarahuka et al. on orthoebolavirus and orthomarburgvirus seroprevalence, and a 2017 review by Bower and Glynn on orthoebolavirus seroprevalence, by better elucidating gaps in research and reasons for variability in previous prevalence findings [19, 20]. Research during or after recent outbreaks of filoviral disease will add to the evidence base reported in these reviews. It is important to update these reviews because of recent large outbreaks, such as the large 2018–2019 EVD outbreak in the Democratic Republic of the Congo, and because of new outbreaks occurring in countries that had previously never experienced outbreaks of such filoviruses, such as the MVD outbreaks that are currently occurring in Equatorial Guinea and Tanzania [7, 8]. In addition, as of 2023, four candidate vaccines are in development for MARV and three candidate vaccines are in development for SUDV [12, 13]. Understanding endemic epidemiology, the role of subclinical infections in transmission, the role of filovirus survivors in transmission, and the role and prevalence of animal reservoirs in spillover events will be important to inform epidemic preparedness, epidemic control methods, design of robust efficacy trials, and the strategy for future vaccine deployment. This is especially important as clinical trials are currently under way in Africa, and design of Phase II vaccine studies should factor in regions that are known to have higher rates of zoonotic spillover of EBOV, MARV, and SUDV.

The strength of this systematic review includes a well-defined search strategy for the primary research question applied to a comprehensive set of databases, with no language or date restrictions. In addition, planned stratification of prevalence results by species, population, assays, and sampling methodology will better explain possible sources of prevalence heterogeneity beyond the scope of what was reported in past reviews. This review will also focus on the assay characteristics used to detect infection because different assays vary in sensitivity and specificity (including varied potentials for cross-reactivity with other viruses), likely making the assay used for diagnosis a central factor in explaining heterogeneity between various prevalence results. Additionally, this review will provide necessary evidence for the development of a conceptual framework to identify gaps in understanding of filovirus epidemiology and transmission. The quality and risk of bias assessment will be useful for developing a conceptual model to illustrate the strength of the body of evidence for understanding prevalence in the context of filovirus outbreaks in sub-Saharan Africa. Using aspects from the JBI Prevalence Critical Appraisal Tool (such as appropriateness of sample frames and validity and reliability of methods used for filovirus detection) and from the GRADE certainty ratings will be central to informing the development of this conceptual framework, as will the Discussion sections of included papers. This will allow for identification and contextualization of the transmission models and root causes of each disease, and aid in identifying existing gaps in research. These root causes and determinants will facilitate the identification of primary causal factors and distal causal factors, ultimately leading to new conceptualizations of possible solutions, interventions, risk factor identification and mitigation, and future research activities.

This review may be limited by heterogeneity between included studies that has also been seen in previous reviews, limiting the potential for meaningful meta-analysis. This review may also be limited by poorly reported methodology, such as sampling strategy or lack of information about the performance and accuracy of the assay used to obtain prevalence. These limitations would represent identified gaps and provide rationale and recommendations for future research.

Filoviruses are among several RNA viruses that cause VHF. The number of filoviral spillover events is rapidly increasing, likely due to globalization, international travel, and climate change. Outbreaks of VHF occur sporadically and irregularly, and their occurrence can be difficult to predict. Understanding prevalence, subclinical infection, and its role in human-to-human transmission will contribute to understanding and controlling increasing numbers of outbreaks.

### Supplementary Information

Supplementary Material 1.

Supplementary Material 2.

## Data Availability

All data were available through a search of either PubMed, Embase, or Web of Science. Most articles used are publicly available, but a few included articles were accessed via a paywall or via an institutional library. The data in the present systematic review and meta-analysis will also be made publicly available.

## Abbreviations

APA: American Psychological Association
BDBV: Bundibugyo virus (species *Orthoebolavirus bundibugyoense*)
BOMV: Bombali virus (species *Orthoebolavirus bombaliense*)
CFR: Case fatality rate
EBOV: Ebola virus (species *Orthoebolavirus zairense*)
ELISA: Enzyme-linked immunosorbent assay
EVD: Ebola virus disease
FANG: Filovirus animal non-clinical group
GRADE: Grading of Recommendations, Assessment, Development, and Evaluations
IgG: Immunoglobulin G
IgM: Immunoglobulin M
JBI: Joanna Briggs Institute
MARV: Marburg virus (species *Orthomarburgvirus marburgense*)
MVD: Marburg virus disease
PCR: Polymerase chain reaction
PICOS: Population, Intervention, Comparison, Outcome, Study Design
PRESS: Peer Review of Electronic Search Strategies
PRISMA-P: Preferred Reporting Items for Systematic Review and Meta-Analysis Protocols
PROSPERO: International Prospective Register of Systematic Review
RAVV: Ravn virus (species *Orthomarburgvirus marburgense*)
RESTV: Reston virus (species *Orthoebolavirus restonense*)
RNA: Ribonucleic acid
RT-PCR: Reverse transcription polymerase chain reaction
SUDV: Sudan virus (species *Orthoebolavirus sudanense*)
TAFV: Taï Forest virus (species *Orthoebolavirus taiense*)
VHF: Viral hemorrhagic fever
