# Supplementary material for: Prevalence of human filovirus infections in sub-Saharan Africa: A systematic review and meta-analysis protocol
Source: Syst Rev. 2024 Aug 15;13:218. doi: 10.1186/s13643-024-02626-w (PMC11325742; doi:10.1186/s13643-024-02626-w)
Supplement: Supplementary file 2 — Supplementary Material 2. [file 13643_2024_2626_MOESM2_ESM.docx]

Article Extraction Form

For each article, fill in the following information. This is for the purposes of creating an Appendix table summarizing the studies that were included in the review.

1. Name of Reviewer
2. APA Citation
3. First Author's Name
4. Year of Publication
5. Institutional/Geographic Afﬁliation(s) of Authors
6. Country/ies of Study
7. Geographical Description of Study
8. Study Design

*Check all that apply.*

Cross-Sectional Study (including prevalence study) Case-Control Study

Cohort Study

Other:

1. Virus Subtypes Detected

*Check all that apply.*

Ebola SUDV Ebola EBOV Ebola RESTV Ebola TAFV Ebola BDBV Marburg MARV Marburg RAVV

Other:

1. Number of People in the Study (n)
2. Age Range of Participants
3. Occupation of Participants

*Check all that apply.*

Miners

Health Care Workers

Otherwise Known to Be Exposed Not Discussed

Other:

1. Other Descriptions of Study Participants
2. Prevalence of Relevant Filovirus(es) Infection (%/proportion and 95% CI if included)
3. Sampling Method

*Check all that apply.*

Simple Random Sampling Systematic Sampling Stratified Sampling Cluster Sampling Convenience Sampling

Voluntary Response Sampling Purposive Sampling

Snowball Sampling

Other:

1. Type of Assay Used

*Check all that apply.*

RT-PCR ELISA

IgG-capture ELISA

Other:

1. Positivity Cutoff and Sensitivity/Specificity Information (if available)

JBI Critical Appraisal/Quality Assessment Form

This form will be used to assess risk of bias and quality of studies included in the review. A table will be made in the Appendix utilizing this information. For a description of each question/criterium, visit this

page: <https://docs.google.com/document/d/1EayPvfGevLxyOUkLsaK9GYulcYdlK2RL/edit>

1. Was the sample frame appropriate to address the target population?

# Mark only one oval.

Yes No

Unclear

Not applicable

1. Were study participants sampled in an appropriate way?

# Mark only one oval.

Yes No

Unclear

Not applicable

1. Was the sample size adequate?

# Mark only one oval.

Yes No

Unclear

Not applicable

Unclear

Not applicable

1. Was the data analysis conducted with sufﬁcient coverage of the identiﬁed sample?

# Mark only one oval.

Yes No Maybe

Not applicable

1. Were valid methods used for the identiﬁcation of the condition?

# Mark only one oval.

Yes No

Unclear

Not applicable

Maybe

Not applicable

1. Was there appropriate statistical analysis?

# Mark only one oval.

Yes No

Unclear

Not applicable

1. Was the response rate adequate, and if not, was the low response managed appropriately?

# Mark only one oval.

Yes No Maybe

Not applicable

This content is neither created nor endorsed by Google.

[Forms](https://www.google.com/forms/about/?utm_source=product&utm_medium=forms_logo&utm_campaign=forms)
